# Operating room first case start times: a metric to assess systems-based practice milestones?

**DOI:** 10.1186/s12909-019-1886-2

**Published:** 2019-12-02

**Authors:** Christopher Ryan Hoffman, Jay Horrow, Shreyas Ranganna, Michael Stuart Green

**Affiliations:** 1 Department of Anesthesiology & Perioperative Medicine, Drexel University College of Medicine, Hahnemann University Hospital, 245 N. 15th Street, Suite 7502, MS 310, Philadelphia, PA 19102 USA; 2 Department of Anesthesiology, Sidney Kimmel Medical College, Thomas Jefferson University Hospital, 111 S. 11th Street, Suite 8490G, Philadelphia, PA USA

**Keywords:** Health resources, Resource allocation, Graduate medical education, Educational measurement, Anesthesia

## Abstract

**Background:**

Resident competence in peri-operative care is a reflection on education and cost-efficiency. Inspecting pre-existing operating room metrics for performance outliers may be a potential solution for assessing competence. Statistical correlation of problematic benchmarks may reveal future opportunities for educational intervention.

**Methods:**

Case-log database review yielded 3071 surgical cases involving residents over the course of 5 years. Surgery anticipated and actual start times were evaluated for delays and residents were assessed using the days of resident training performed at the time of each corresponding case. Other variables recorded included day of week, attending anesthesiologist name, attending surgeon name, patient age, sex, American Society of Anesthesiologists physical status classification (ASA PS), and in-patient versus day surgery status. Mixed-effect, multi-variable, linear regression determined independent determinants of delay time.

**Results:**

The analysis identified day of the week (F = 25.65, *P* < 0.0001), days of training (F = 8.39, *P* = 0.0038), attending surgeon (F = 2.67, *P* < 0.0001), and anesthesiology resident (F = 1.67, *P* = 0.0012) as independent predictors of delay time for first-start cases, with an overall regression model F = 3.09, r^2^ = 0.186, and *P* < 0.0001.

**Conclusions:**

The day of the week and attending surgeon demonstrated significant impact of case delay compared to resident days trained. If a learning curve for first-case start punctuality exists for anesthesiology residents, it is subtle and irrelevant to operating room efficiency. The regression model accounted for only 19% of the variability in the outcome of delay time, indicating a multitude of additional unidentified factors contributing to operating room efficiency.

## Background

The importance of efficiency in health care becomes most evident in the operating room. Punctuality and minimizing delays play key roles in efficient because time is the most costly operating room resource. Cost estimates range from $20 per minute when excluding personnel to $30 to $80 per minute when including physician and nursing staff [1].

An efficient operating room starts the day on time. Delays in the first case start time could be a measure of resident competency in peri-operative care, and of a teaching hospital’s efficiency. Preoperative delays due to resident training level affect surgeons and other perioperative staff.

Providing high-quality resident education alongside an efficient cost-effective operating room presents a challenging problem for administrators, educators, and trainees. Disproportionate emphasis on efficiency may compromise resident education and vice versa [2]. The anesthesiologist controls several determinants of operating room efficiency [3]. Examining the effect resident training has on metrics of efficiency can provide administrators and educators with tools to gauge effectiveness of resident education as well as cost utilization. Since the competency of practicing anesthesiologists is evaluated in part through their efficiency, a similar principle could apply to resident education. Residents that prove to be outliers regarding efficiency, when compared to the expected times for their training level, could receive additional coaching to prepare them for the rapid pace of everyday practice. Establishing benchmarks in these metrics may help educators and program directors identify residents that may be lagging behind a standard service guarantee. If a pattern reflecting resident competency and relevant impact toward operating room efficiency cannot be identified, this would warrant addressing the means to which administrators and educators assess competency. The emphasis placed on the perception that residents cause delays or impact operating room function would have to be allocated elsewhere and judgment of resident performance reimagined.

This exercise probed the determinants of first case delays in start time, with a focus on resident training experience. It hypothesized that delays decreased with more training, with an eye to using this metric to assess systems-based practice competency of anesthesiology residents.

## Methods

This retrospective, observational study polled the case-log database maintained by a tertiary care facility with 2 units in close proximity sharing personnel, one exclusively for day surgery, the other for mixed in-patient and day surgery cases. The Drexel University Institution Review Board (IRB) approved this retrospective review. The first scheduled (non-emergency) adult case of every non-holiday weekday (Monday through Friday) at every anesthetizing location in the 2 units (i.e., no “off-site” locations) from July 1, 2013 through February 28, 2018 constituted the initial observations. Information extracted from the database consisted of date, day of week, anesthesiology resident name, attending anesthesiologist name, attending surgeon name, patient age, sex, American Society of Anesthesiologists physical status classification (ASA PS), in-patient versus day surgery status, and time the patient entered the operating room. Only cases of ASA physical status 1, 2, or 3 patients involving an anesthesia resident and attending anesthesiologist were retained. Cases during CA-1 orientation (first 6 weeks of residency) were included. Transplant, intrathoracic, obstetric, neurosurgical, and otolaryngological procedures were excluded.

We calculated days of training as the number of days elapsed between the start date of an individual resident’s training and the date of operation, and the outcome variable “delay time” as the number of minutes elapsed from our service guarantee start time of 0730 h for Mondays, Thursdays, and Fridays, or 0815 h for Tuesdays and Wednesdays, and the clock time noted for patient entering the operating room. Negative values for delay time occurred if patients entered the operating room prior to the service guarantee time. A series of diagnostic data checks of non-outcome fields sought data-entry errors.

Following data cleaning, Pearson correlation coefficients for the pairwise combinations of the 3 numerical predictors, variance inflation factors, and condition numbers assessed potential multicollinearity [4]. Variables with r^2^ > 0.50 or variance inflation factor > 50 were considered for collinearity. Remaining predictors entered a mixed-effects, multi-variable, linear regression to determine independent determinants of delay time. Predictors with significance levels < 0.05 constituted the final model, following examination of regression diagnostics. Descriptive statistics of the data utilize mean, standard deviation, median, and range. Where appropriate, interquartile range is also reported. SAS version 9.0 (Cary, NC) conducted the analysis.

## Results

The database poll yielded 3076 observations. Data cleaning deleted 5 observations naming unrecognized individuals: 2 different unknown attending anesthesiologists and 3 unnamed attending surgeons. The distribution of observations by resident level of training was 62% CA-1, 18% CA-2, and 20% CA-3, reflecting the dearth of sub-specialty and off-site rotations in the first year of training.

The 3071 cases involved 59 individual anesthesiology residents, 32 attending anesthesiologists, and 115 attending surgeons. The 59 anesthesiology residents had completed 369 ± 318 (SD) days of training, with range 0–1092 and quartiles at 106, 251, and 621 days. They participated in 52 ± 27 first-start cases (median 57, range 5–111). The 32 anesthesiology attendings had supervised 96 ± 60 first-start cases with those residents (median 85, range 9–249). A majority (*n* = 60) of the 115 attending surgeons, participated in fewer than 10 first-case starts.

The 3071 patients comprised 945 in-patients (31%) and 2126 day-surgery patients (69%) aged 49 ± 13.9 years (range 18–75, median 51); 52.7% were women. ASA physical status (PS), distributed as 9% PS 1, 52% PS 2, 39% PS 3, did not differ by CA-level of training (χ2 with 4 degrees of freedom = 5.92, *P* = 0.21). CA-1 s cared less frequently for in-patients (28% of CA-1 patient cohort v 37% for CA-2 and 34% for CA-3; χ2 with 2 degrees of freedom = 20.3, *P* < 0.0001).

The outcome variable, delay time, ranged from 82 min early to 116 min late, with mean 4.9 ± 15.9 mins late, median 0, and mode 0.

Predictor variables for the multi-variable linear regression were identities of the resident, attending anesthesiologist, and attending surgeon, days of resident training, day of the week, patient ASA physical status, and in-patient/day surgery status. Multicollinearity tests did not exclude any predictors. The analysis identified day of the week (F = 25.65, *P* < 0.0001), days of training (F = 8.39, *P* = 0.0038), attending surgeon (F = 2.67, *P* < 0.0001), and anesthesiology resident (F = 1.67, *P* = 0.0012) as independent predictors of delay time for first-start cases, with an overall regression model F = 3.09, r^2^ = 0.186, and *P* < 0.0001.

A sensitivity analysis identified the individual contributions of each anesthesiology resident to delay time. Three senior residents near the end of their training had excessive average delays (29.9, 11.7, 9.0 min) but few first-case starts (5, 27, 23 respectively). A sensitivity analysis with these 3 residents and their 55 cases deleted yielded the same independent predictors with similar results (days of training *P* = 0.0029).

Another sensitivity analysis attempted to identify particular residents for whom training days impacted first-case delay time by inserting an interaction term (days training * resident name). Those results showed loss of significance for the individual variables “resident” (*P* = 0.063) and “days training” (*P* = 0.77), with the interaction term significance at *P* = 0.023.

In the original model, for every 100 days of training, the delay time shortened by 0.35 ± 0.12 (SE) minutes, or 21 ± 7 s. Table 1 presents the calculated least square means from the regression for continuous predictor variables.

**Table 1:** Least squares means for predictor variables

Predictor	Delay (minutes)
Day of week	
Monday	6.33
Tuesday	6.89
Wednesday	−2.24
Thursday	11.23
Friday	6.33
ASA PS	
1	4.89
2	5.94
3	6.30
Patient status	
In-patient	5.75
Day-surgery	5.67

## Discussion

This study observed seven predictor variables and their relationships with workflow efficiency as defined by scheduled first cases beginning in a timely manner. The day of the week exhibited the highest impact (F = 25.65, *P* < 0.0001). The days of the week that start 45 min later due to anesthesia and surgery conference time performed best. This may be due to the increased time patients have to arrive and undergo processing by preoperative nursing staff. If so, then this suggests a target for improvement, though efficiency in this particular aspect would not be anesthesia-related.

The attending surgeon was another strong factor (F = 2.67, *P* < 0.0001). Possible explanations include surgeon punctuality, degree of utilization of preadmission testing facilities, patient population co-morbidities, and complexity of room preparation associated with particular surgeons.

Days of training showed a trivial (21 s per 100 days training) yet statistically significant association (*P* = 0.0038), the significance likely due to the overpowered analysis. If a learning curve for first-case start punctuality exists for anesthesiology residents, it is subtle and irrelevant to operating room efficiency. Individual anesthesia resident, a weak determinant, did not retain significance in the sensitivity analysis, suggesting a spurious significance of both days of training and anesthesiology resident identity by the original regression.

Three variables of interest lacked statistical significance: the attending anesthesiologist, the extent of patient systemic disease as defined by ASA PS, and inpatient/ambulatory case. Some veteran operating room personnel may find these results surprising, underscoring the value of collecting and analyzing data to examine our individual assumptions and biases.

This study excluded ASA PS 4 and 5 patients as well as some types of surgery (transplant, cardiac, intrathoracic, obstetric, neurosurgical, or otolaryngological procedures). These exclusions attempted to control for outliers of delay due to complicated room setup, extensive patient evaluation, or patient stabilization.

The regression model accounted for only 19% of the variability in the outcome of delay time. Thus, there are other unidentified or unmeasured factors not in the model. Patient-related issues that may contribute to delay include poor punctuality, incomplete preadmission testing, language barriers prolonging preoperative assessment and informed consent, and difficult venous cannulation. Preoperative nurse staffing ratios and general varying work efficiency and intraoperative nursing room or equipment preparation may slow progression to the operating room. This retrospective study could not totally exclude external factors requiring extra preparation. The retrospective design did remove the possibility of personnel working more efficiently under observation: they were not aware start times were being analyzed. Future analyses can include additional factors to account for some of the unassigned 81% of variation.

First case delay statistics can help manage inter-departmental and patient expectations. Late case starts increase anxiety for both patients and their families, and can lengthen the work day, and increase staff dissatisfaction [5, 6]. While starting a case on-time may not necessarily free enough time to schedule an additional case, it does allow less rush at the beginning; rush contributes to an unsafe working environment [7].

The ACGME views “facilitating cost-effective and safe anesthesia care” as part of the systems-based practice milestone when evaluating resident competency [8]. Consensus on how to evaluate this remains elusive. This evaluation of how resident training affects efficiency in an academic facility introduces the possibility of using perioperative metrics as a way to monitor milestone progression. Results were surprising: major factors were anesthesiology-independent, and the resident training impact was trivial. First case delay did not reflect anesthesiology resident progress, and thus is not suitable to assess systems-based practice competency for anesthesiology residents.

## Limitations

The results apply only to the one program evaluated. A larger scale review of many anesthesia providers caring for patients with different ranges in comorbidities may elicit different results. The design excluded ASA PS 4 and 5 patients to prevent that small minority of cases from exerting undue influence on the regression. While doing so potentially prevents the regression from detecting increasing efficiency of residents in preparing these sicker patients for surgery, it also prevents an inappropriate influence of the few such cases assigned to residents early in training. The design also excluded patients undergoing transplant, cardiac, intrathoracic, obstetric, neurosurgical, and otolaryngological procedures. While each of these entails special circumstances causing potential delays beyond control of anesthesiology residents, the omission might have masked a special improvement of resident efficiency with days of training for these cases. The retrospective design cannot validate data collection accuracy at the time of entry. Procedure times entered haphazardly for any reason (e.g. workflow pressure, indolence, or haste) may result in imperfection.

## Conclusions

Days of anesthesiology residency training had a trivial impact on operating room first case start times, suggesting that those times cannot form an appropriate metric for systems-based practice competency for anesthesiology residents.

## Data Availability

The datasets used and/or analyzed during the current study are available from the corresponding author on reasonable request.

## Abbreviations

ACGME: Accreditation Council for Graduate Medical Education
ASA: American Society of Anesthesiologists
CA: Clinical Anesthesia
IRB: Institutional Review Board
PS: Physical Status
SD: Standard Deviation
